# Experimental investigation of the elasticity of the human diaphragm

**DOI:** 10.1186/1471-2482-10-5

**Published:** 2010-01-30

**Authors:** Gerhard Steinau, Christian Hohl, Andreas Prescher, Daniel Kaemmer, Gabriele Böhm

**Affiliations:** 1 Department of Surgery, University Hospital Aachen Pauwellsstr 30, 52074 Aachen, Germany; 2 Department of Diagnostic and Interventional Radiology HELIOS Hospital Siegburg, Ringstr 49, 53721 Siegburg, Germany; 3 Institute for Anatomy, University Hospital Aachen, Pauwelsstr. 30, 52074 Aachen, Germany

## Abstract

**Background:**

Traumatic diaphragmatic ruptures affect mainly the left side. In an experimental study in human corpses we examined the stretch behaviour of the left and right diaphragmatic halves.

**Methods:**

In a total of 8 male and 8 female corpses each diaphragmatic half was divided into 4 different segments. Each segments stretch behaviour was investigated. In steps of 2 N the stretch was increased up to 24 N.

**Results:**

In the female the left diaphragm showed a stronger elasticity compared to the right. Additionally the left diaphragm in females showed a higher elasticity in comparison to the left in males. Traumatic diaphragmatic ruptures affect mostly the central tendineous part or the junction between tendineous and muscular part of the diaphragmatic muscle. Accordingly we found a lower elasticity in these parts compared with the other diaphragmatic segments.

**Conclusion:**

In summary it can be said that albeit some restrictions we were able to determine the elasticity of different diaphragmatic segments quantitatively and reproduceably with our presented method. Thereby a comparison of results of different diaphragmatic segments as well as of both diaphragmatic halves and of both genders was possible

## Background

Diaphragmatic ruptures arise from blunt trauma. They appear predominantly on the left side [1,2]. A hypothesis for this phenomenon is seen in the protected position of the right diaphragm above the liver. No investigations exist as to whether the preference for the left side could not be caused by a different stretch behaviour of the two diaphragm halves.

Since tears mainly occur within the Centrum tendineum or at the junction of tendineous and muscular part of the diaphragm [3], our investigations in corpses focussed especially on the stretch behaviour of the centrum tendineum, the junction between tendineous and muscular part and the connection between diaphragm and the thoracic wall. If the traumatic force mainly involves the thoracic wall it may also disrupt the diaphragm at its costal or vertebral insertions [2,4]. Up to now comparative investigations have not been described in the literature.

## Methods

**Figure 1:**
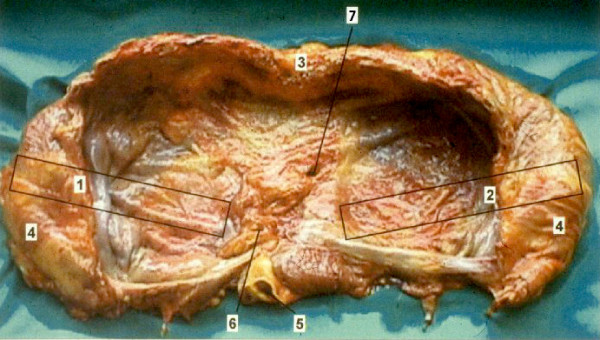
**Excised diaphragm from above**. 1. Excised part for left segment of diaphragm; 2. Excised part for right segment of diaphragm;3. Sternum; 4. Ribs; 5. Aorta; 6. Hiatus oesophagus; 7. Foramen venae cavae

For this project we investigated human corpses. These persons while still alive had kindly agreed to place their corpses after death at the disposal of the University Department of Anatomy according to ethical and legal standards. The diaphragms of 8 male and 8 female corpses were investigated. The age of the male corpses ranged from 45 to 78 years, of the females from 46 to 79 years. The delay of time between death and investigation of the diaphragm for the male ranged between 12 and 85 hours, for the female between 12 and 120 hours. No macroscopic visible changes of the diaphragms were present, and no systemic illness potentially affecting the mechanical function of the diaphragm were known (Figure 1)

### Experimental set-up

**Figure 2:**
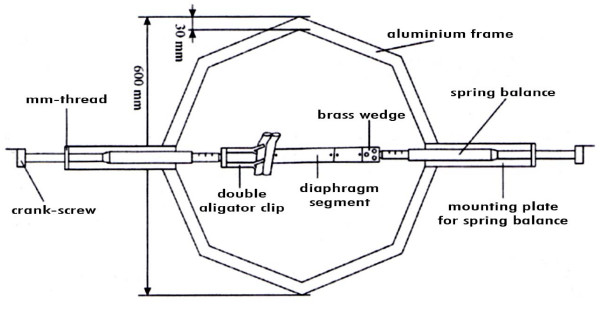
**Experimental set up**.

The measuring device consisted of an octagonal 3-cm-wide aluminium frame. Inbuilt in each inner side of this octagon were holding devices to which spring-balances were connected. Two spring-balance fixtures were adjustable in their lengths and could be used as measurement devices for smaller structures. The whole construction stood on four height-adjustable base feet. Figure 2 shows a pattern of the rack (Figure 2).

### Experimental sequence

**Figure 3:**
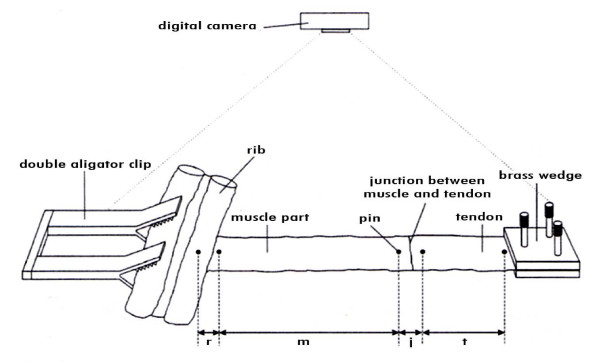
**Diaphragmatic segment in detail**. For evaluation defined segments: r: rib insertion; m: Muscular segment (part of the costal area); j: Junction of muscle and tendon; t: Tendon part (part of central ares)

Out of every diaphragmatic half a 3-cm-wide stripe was excised. At one end of this stripe the muscle inserted into two neighbouring ribs. In the following text we call this part of the stripe 'rib insertion'. At the other end of the diaphragmatic stripe was the centrum tendineum. As demonstrated in Figure 3 the stripe was segmented into 4 parts: segment one were the ribs, segment two was muscle, segment three was the junction of muscle and tendineous part, segment four the centrum tendineum (Figure 3).

The used measuring devices were commercial mechanical spring-balances with a maximum tractive power of 25 N and a feather way of 10 cm. The exactness amounted to 1% of the maximum permissible load according to manufacturer's specifications

### Investigational procedure

The stretch of the stripes took place through the crank screws and the force transmitted onto the stripes could be read on the spring-balances. In steps of 2 N the strength effect was increased. At 2 N the first digital photo of the stripe was made. The used digital camera had a resolution of 640 × 480 pixels. By means of a tripod the camera was brought in its position above the work station. The distance between stripe and camera amounted to 45 cm. The investigation was finished at the premature rupture of the stripe or when 24 N were accomplished.

### Analysis

Purpose of the experiment was to record the stretch behaviour of the different diaphragm stripes. We measured the proportional strech during constantly increasing traction force. Linear regression analysis of these proportional stretch steps resulted in a certain stretch value specific for each segment of the stripe. Microsoft Excel 97 was used for the analysis.

Evaluation of the data followed Hooke's law: F = -k·delta L. (F = force, k = spring constant, delta L = distance). The strech value sv is defined as the reciprocal value of the spring constant k.

During the experiment the length of a segment was displayed by the distance of pins or markers. By means of their co-ordinates on the digital photo the length of each segment was measured in pixel for every stretch step. Because the measurement began with 2 N, the length of the segments at 0 N was extrapolated with Excel. With this extrapolated output length we calculated the proportional stretch for every stretch step. For the analysis of the strech behaviour of the diaphragm stripes, we plotted the length increase against the traction force displayed on the balances. Using the linear regression method we calculated the slope of the graph representing the strech value sv = 1/k. For the characterization of the stretch behaviour of each segment we therefore evaluated stretch curve and a stretch value in each case.

## Results

### Stretch values in comparison

The stretch value of all 16 investigations were evaluated separately according to diaphragmatic segment, sex and side localisation. 8 measured values per sub-group were available for the determination of the mean and standard deviation. For statistical analysis of the difference in stretch behaviour between left and right diaphragm we used the linked Wilcoxon test.

The average of the stretch values 10^-5 ^mN^-1 ^can be seen in table 1.

**Table 1:** The average of the stretch values (10^-5^ mN^-1^)

***Males:***				
	**rib**	**muscle**	**junction**	**tendon**
				
left:	34,0	18,4	12,3	9,8
right:	34,6	22,2	11,8	11,2
				
***Females***				
	**rib**	**muscle**	**junction**	**tendon**
				
left	50,3	27,5	17,0	12,2
right	39,2	23,5	10,2	9,0

### Male diaphragms

Measurements in all right and left stripes took place up to 24 Newton. Up to this point no tears were observed nor could we detect any signs of weakness predicting a forthcoming tear. In one right stripe the measured value taken at 24 N was not taken for evaluation. Here the stripe stretched with rising charge of 22 to 24 Newton so strongly that a measuring artefact was supposed on grounds of an imminent tear.

**Figure 4:**
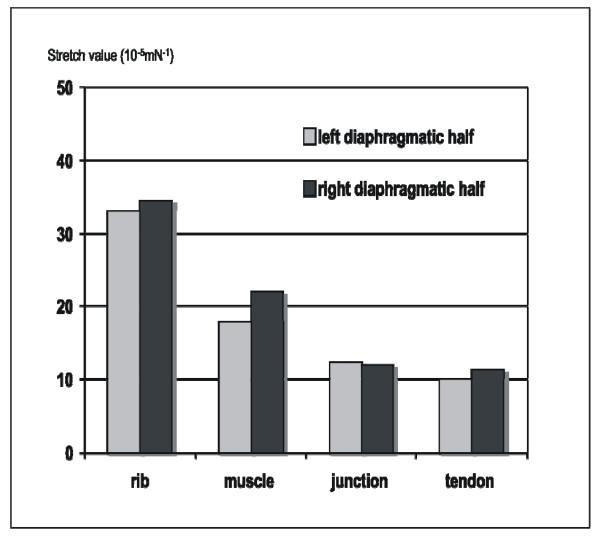
**Stretch values for diaphragmatic segments in males**.

The highest elasticity was observed at the rib insertion in 15 stripes (in 8 of the left and 7 of the right side). The next best elasticity was seen in the muscle part of 14 stripes. Less elasticity was seen at the junction of muscle and tendon. In all examined stripes the tendineous segment showed the least elasticity (Figure 4).

With many stripes of both diaphragmatic sides we observed a 2-phase stretch behaviour, particularly for the rib insertion as well as the muscular part. At the beginning of the stretch these parts showed a higher elasticity. During further stretching a second phase was observed where no increase in elasticity was observed, rather a stagnation.

### Female diaphragms

In total 16 diaphragms (8 right and 8 left halves) were examined. With three of the examined diaphragm stripes (1 of the right, 2 of the left side) an exceedingly strong increase of the elasticity was observed. This occurred in 2 stripes at the increase from 20 to 22 N and in the other at the increase from 22 to 24 N. In the latter the diaphragm tore at 24 N. With all other investigated stripes the measurements could be carried out up to 24 N.

**Figure 5:**
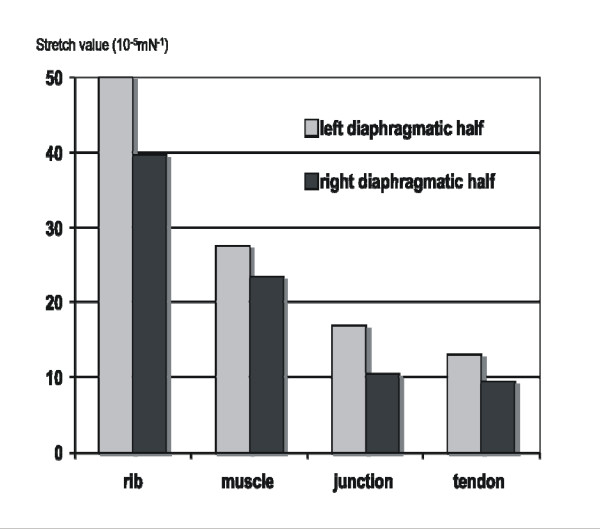
**Stretch values for diaphragmatic segments in females**.

The most elastic part was the rib insertion in all but three stripes, the muscular segment was only slightly elastic, followed by the junction between muscle and tendon. The most rigid part again was the tendineous part. (Figure 5).

In contrast to the male stripes a 2-phase stretch behaviour could seldom be observed with the female stripes in the according segments. In the female case we found a near linear stretch behaviour in most stripes of the four segments.

### Comparison of female and male diaphragmatic stripes

**Figure 6:**
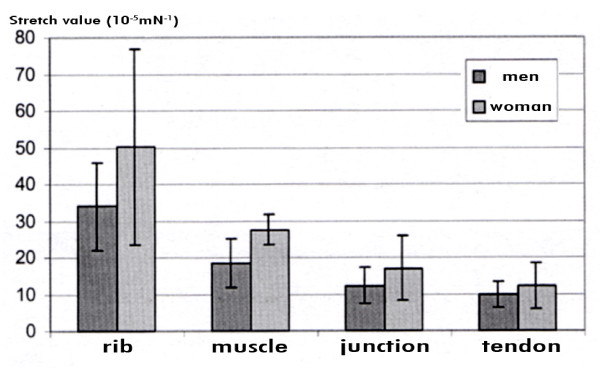
**Stretch values for left diaphragmatic half in males and females**.

The comparison of the average stretch values of left diaphragmatic halves in women with those in men shows that all four female diaphragmatic segments are more elastic than the according male segments (Figure 6). The rib insertion segment in the female is 48.2% more elastic than in the male. For the muscular segment the elasticity lies accordingly at 49.8% higher in the female, for the junction of muscle and tendon at 37.8% and for the tendineous segment at 24.3%.

**Figure 7:**
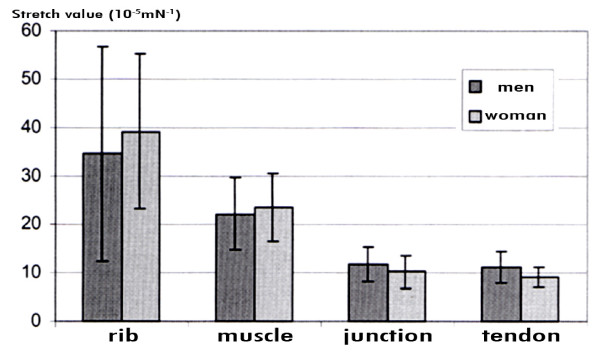
S**tretch values for right diaphragmatic half in males and females**.

The right diaphragmatic halves don't show these differences. The female rib insertion segment is 13.5% more elastic than in the male, the muscular segment 6.1% (Figure 7). The difference is altogether less prominent compared to the left diaphragm. For the junction segment and the tendon segment the male stripes show a higher elasticity. In the male the latter show a higher elasticity by 16.3% and 24.4% respectively.

### Comparison of left and right diaphragmatic halves

**Figure 8:**
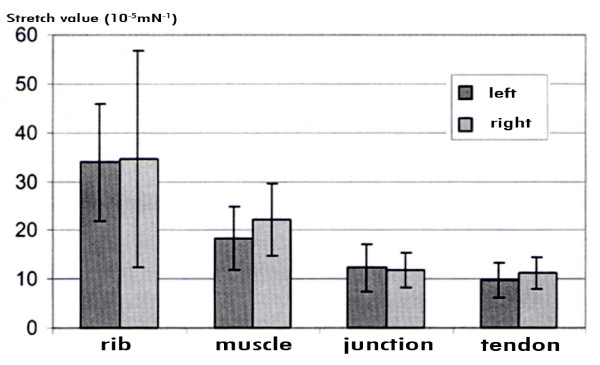
**Stretch values for diaphragmatic segments in males**.

If one compares the stretch average values in the male left and right diaphragmatic half, the muscular segment of the right side is 14.6% more elastic. The rib insertion and junctional segments of both sides have similar elasticity, with stretch differences being less than 5%. The rib insertion segment is with 1.8% more elastic on the right side, the junctional segment is with 4.3% more elastic on the left side (Figure 8).

In the female all four segments on the left side show a higher elasticity compared to the right. The rib insertion segment is by 28.3% more elastic on the left than on the right, the muscular segment by 16.9%, the junctional segment by 67.1% and the tendineous segment by 34.9%. A statistically significant difference could neither be found when comparing right and left diaphragmatic stripes in male or female nor when comparing according segments in male and female.

## Discussion

Possible causes for a diaphragmatic rupture are direct (penetrating and shot injuries) as well as indirect (traffic accidents, fall from great height and blunt trauma) forces. 75% of all diaphragmatic injuries are caused by blunt traumas, 25% by penetrating injuries [2,5]. Most cases of blunt traumas lead to a massive wide force on the abdominal and thoracic cavity. The result is an acute increase in intraabdominal pressure exerting its forces on a reflectively contracted diaphragm. When the pressure rises to a certain value the elastic limit is reached and this causes the indirect diaphragmatic rupture [1].

According to calculations by Sauer and Lutz in 1976 this sudden increase of intraabdominal pressure has to reach more than 100 mm Hg in order to cause rupture [6]. As a rule this leads to a tear within the centrum tendineum or within the junctional segment of the diaphragm [2,5]. If the traumatic force on the other hand mainly hits the thoracic wall, the destruction of the diaphragm is due to the snap back of the compressed diaphragm resulting in a tear at the costal and vertebral insertions [2,4].

The present experimental set-up was developed to measure the elasticity of the human diaphragm quantitatively. Up to now only stamp compression tests on rats have been described in the literature [7]. With the latter no answers could be given as to what mechanical qualities different diaphragmatic segments have. Therefore, the development of a new experimental concept was necessary.

It also would have been interesting to put one fixation point at the sternum. However, many corpses had a previous thoracotomy and would not have been suitable. In order to keep a certain corpse number we rejected this idea. Consequently neither the triangle of Bochdalek nor of Larrey-Morgagni, which count as locus minoris resistentiae, were included in the examined diaphragmatic parts. We also abandoned the idea of a lumbar fixation since its construction would have meant an enormous effort with little promise of significant outcome.

By this restriction on a well functioning measuring distance we were able to increase the stretch tension to a maximum value of 24 N. This maximum value was orientated on the elasticity measurements of the abdominal wall [7,8] and turned out to be a convenient parameter.

The sex-specific comparison of the mean stretch values showed a higher elasticity for the rib insertion segments and the muscular segments in women. The muscular part of the measured female diaphragms was always thinner compared to the male, and as a rule has a lower surface value in the female. This leads to an increased elasticity in the female.

Because of the biologically given diaphragmatic thickness the female muscular part and the female rib insertion zone are more flexible than the according male segments. Whether the latter is only due to the thickness of the muscle or whether other tissue qualities play a role, cannot be said at present.

The comparison of the outcome with regard to the sexes only showed a higher elasticity in two female segments on the left side of diaphragm (junctional zone and tendinuous part). On the right side, however, these segments showed a higher elasticity in men.

Within the applied range of forces two ruptures at the rib insertion of muscle occurred, both times in female corpses. Nevertheless, in the literature diaphragmatic ruptures are more frequently described in men [2]. In general may be due to the way the force is applied in our experiments that this rather leads to a disruption at the rib insertion than to a tearing within the tendineous part or at the junction of tendon and muscle. Within clinical context it is often a pressure load exerting the force and this leads mainly to tears within the latter two areas [3,9]. We assume therefore that the female muscle insertion at the rib more easily withstands pressure than a tearing force.

Since an acute increase in intraabdominal pressure is the main cause of diaphragmatic rupture [1,2] the defect manifests itself rather within the tendon or at the junction zone. Due to this type of force in clinical practice the increased tendency of tearing at the rib insertion in our experimental setting with tearing rather than pressure force being exerted would therefore in real life be less likely.

Because in men the muscular diaphragmatic area shows a greater thickness this could be a reason for more frequent tearing within the centrum tendineum or the transitional area in men compared to women. Important for the pathogenetic mechanism is that the force of increased abdominal pressure hits the contracted diaphragm. In the case of greater muscle mass in cross section a bigger tension would be built up, so that this area is more likely to tear [1,2,10]

The explanatory power of the investigations is limited by the absence of muscle tone during the stretch measurements which certainly influences the elasticity. Moreover the diaphragm is shaped three-dimensionally like a dome and this is not taken into account by the linear stretch measurements in our setting. Besides, it is unclear whether the area well-chosen for the measurements can be representative for the whole diaphragm and whether the effect of the measured tearing forces are comparable with the effect of a traumatic pressure force.

Because the tearing that follows a traumatic diaphragmatic rupture mostly occurs in the centrum tendineum or within the junctional zone [2,5], we particularly examined these two segments for conspicuities in their stretch behaviour. Both segments showed a lower elasticity than the remaining examined segments of the diaphragm. Also no ruptures appeared within these areas during our investigations. A possible explanation of this contradiction could be the absent muscle tone.

The rarer rupture at the costal or lumbar insertion rather occurs with thoracic traumas. Here the disruption of the diaphragm occurs due to the sudden springing back of the beforehand compressed diaphragmatic aperture [4]. Our own experiments rarely showed a rupture, and if so its localisation was always observed at the rib insertion. The rib insertion segment also owned a substantially higher elasticity compared to the other segments. The applied tearing force rather leads to a disruption at the rib insertion than to a tearing of the other examined segments. This conclusion agrees with the pathophysiological mechanism described by Schafmayer where a tearing force leads to rupture at the rib insertion.

## Conclusions

In summary it can be said that albeit some restrictions we were able to determine the elasticity of different diaphragmatic segments quantitatively and reproduceably with the above presented method. Thereby a comparison of results of different diaphragmatic segments as well as of both diaphragmatic halves and of both genders was possible

## Competing interests

The authors declare that they have no competing interests.

## Authors' contributions

GS and GB have made substantial contributions to conception and design. GB and AP have been involved in revising the manuscript critically for important intellectual content. GS has made substantial contributions to acquisition of data. CH and DK have been involved in analysis and interpretation of data and GS has given final approach of the version to be published. All authors read and approved the final manuscript.

## Pre-publication history

The pre-publication history for this paper can be accessed here:

http://www.biomedcentral.com/1471-2482/10/5/prepub
